# Cognitive behavioural therapy self-help intervention preferences among informal caregivers of adults with chronic kidney disease: an online cross-sectional survey

**DOI:** 10.1186/s12882-022-03052-7

**Published:** 2023-01-04

**Authors:** Chelsea Coumoundouros, Paul Farrand, Alexander Hamilton, Louise von Essen, Robbert Sanderman, Joanne Woodford

**Affiliations:** 1 Department of Women’s and Children’s Health, Healthcare Sciences and E-Health, Uppsala University, Uppsala, Sweden; 2 Clinical Education, Development and Research (CEDAR), Psychology, University of Exeter, Exeter, UK; 3 Faculty of Health and Life Sciences, University of Exeter Medical School, University of Exeter, Exeter, UK; 4 Exeter Kidney Unit, Royal Devon University Healthcare NHS Foundation Trust, Exeter, UK; 5 Department of Health Psychology, University Medical Center Groningen, University of Groningen, Groningen, the Netherlands

**Keywords:** Informal caregiver, Online survey, Self-help, Cognitive behavioural therapy, Mental health, Chronic kidney disease, Intervention preferences

## Abstract

**Background:**

Informal caregivers (i.e. family and friends) provide essential support to people with chronic kidney disease (CKD). Many informal caregivers experience mental health problems such as anxiety and depression due to the caregiving role, and commonly have unmet psychological support needs. One potential solution is cognitive behavioural therapy (CBT) self-help interventions that are less reliant on extensive involvement of healthcare professionals, which may increase access. Within the intervention development phase of the MRC framework, the study’s primary objective was to examine informal caregivers’ self-help intervention preferences (e.g. delivery format, content). Secondary objectives were to describe the informal caregiver’s situation (e.g. type of care activities) and mental health (symptoms of depression, anxiety, and stress).

**Methods:**

An online cross-sectional survey conducted in the United Kingdom. Informal caregivers of adults living with CKD were recruited via social media, websites, newsletters, magazine articles, a podcast episode, and paid Facebook advertisements. The survey examined: informal caregiver characteristics; care recipient characteristics; self-help intervention preferences; and informal caregiver’s mental health using the DASS-21. Data were analysed using descriptive statistics.

**Results:**

Sixty-five informal caregivers participated. The majority (85%) were female, caring for a male (77%) spouse/partner (74%). Responses indicated 58% of informal caregivers were experiencing at least mild depression. In total, 48% indicated they were likely to use a CBT self-help intervention, preferring an intervention provided via internet (e.g. website) (64%), workbook (56%), or individually in-person (54%). Regarding content, interventions should cover a wide range of topics including living with CKD, support services, informal caregiver’s physical health, and diet. Overall, 48% reported a preference for a supported intervention, with support delivered in-person or via email by a trained professional at a community organisation.

**Conclusions:**

Results suggest CBT self-help interventions may be an acceptable way to provide psychological support to informal caregivers, however the study is limited by the small sample size. A wide range of intervention preferences were identified indicating a need to tailor intervention content and delivery to enhance acceptability and engagement. Results will inform development of a CBT self-help intervention for informal caregivers of people with CKD.

**Supplementary Information:**

The online version contains supplementary material available at 10.1186/s12882-022-03052-7.

## Background

Chronic kidney disease (CKD) has a significant impact on people living with CKD, their informal caregivers (hereafter referred to as caregivers), and the health and social care system [1, 2]. CKD has an approximate prevalence of 7% in England [3, 4], and between 11 and 13% globally [5]. This is anticipated to rise as both the proportion of older adults and those living with health conditions which can lead to CKD (e.g. diabetes and obesity) increase [1, 3]. Where CKD progresses to end-stage kidney disease, kidney replacement therapy, such as dialysis or a kidney transplant, may be required [6]. Kidney replacement therapies make up the majority of costs placed on the healthcare system related to CKD [7], and place significant burden on the person receiving kidney replacement therapy and their caregivers (i.e. family members and friends) [8–10].

Caregivers of people with CKD may take on a variety of responsibilities, such as helping with household tasks, assisting with medical treatments and appointments, supporting lifestyle changes (e.g. dietary restrictions), and providing emotional support [8, 11]. Caregivers can also be coping with their own physical health conditions while providing informal care [12, 13]. Impacts associated with the provision of informal care are recognised by the Standardised Outcomes in Nephrology core outcome sets [14], with caregivers commonly experiencing depression, anxiety, caregiver burden, and poor quality of life [8, 15–17]. Caregiver mental health can also impact the mental health of the person with CKD [18, 19].

Despite the need for psychological support among caregivers of people with CKD, little research has been conducted to develop psychological interventions to support caregivers' mental health needs [20–22]. Lack of research on caregivers of people with CKD is in stark contrast to research for other caregiver groups, with 332 interventions identified for dementia caregivers in a recent meta-analysis [23]. In the United Kingdom (UK), limited psychological support is available within kidney care units, however availability of support services and staff (e.g. psychologists, social workers) varies across units and is often lacking [24]. It is also unclear to what extent this support is accessible to caregivers themselves, and whether available support aligns with caregivers’ needs and preferences.

One promising solution to address caregivers’ need for psychological support is self-help interventions. Self-help interventions can increase access to psychological support as they are less reliant on extensive involvement of healthcare professionals [25, 26]. Furthermore, delivery through a variety of formats including via the internet or smartphone applications, can help mitigate common barriers to accessing mental health interventions such as stigma and lack of time [26–29]. One type of evidence based self-help intervention is cognitive behavioural therapy (CBT) self-help [30–32]. While the core elements associated with effectiveness of CBT self-help are established [33], tailoring CBT self-help interventions to the needs and preferences of specific user populations is needed to ensure intervention acceptability in practice [34–36].

The new Medical Research Council (MRC) framework for developing and evaluating complex interventions [37] will be followed to develop an effective and acceptable CBT self-help intervention tailored for caregivers of people with CKD, while considering implementation context and stakeholder engagement during intervention development [37–39]. Intervention development involves a number of actions which are outlined in the intervention development framework developed by O’Cathain et al. [39]. In relation to this framework [39], this study begins to address intervention development actions such as, primary data collection, designing the intervention by generating ideas regarding intervention content, format and delivery, and understanding context. Context is a broad concept referring to the factors that surround and influence an intervention such as the implementation setting, political environment, and user needs and preferences [40]. Exploring elements of context during the development of a CBT self-help intervention may facilitate intervention tailoring to enhance acceptability, and should increase future implementation potential [39].

### Research aim & objectives

The overall aim was to examine contextual factors related to caregivers to inform development of a CBT self-help intervention to support the mental health of caregivers of people with CKD. The primary objective was to examine caregivers’ intervention preferences (e.g. delivery format, content). The secondary objectives were to describe caregivers’ situations (e.g. what type of care activities they do, who they care for), and mental health (symptoms of depression, anxiety and stress).

## Methods

### Design

An anonymous, online, cross-sectional survey was hosted via Qualtrics (Qualtrics, Provo, UT), with results reported following the Checklist for Reporting Results of Internet E-Surveys (Additional file 1) [41]. The term care recipient is used to refer to the person with CKD who is receiving informal care.

### Participants

Individuals were eligible to participate if they: (1) were an adult (aged 18 years or older); (2) lived in the United Kingdom (UK); (3) self-identified as a caregiver currently providing unpaid care and support to an adult with CKD; and (4) were able to read and write English.

### Recruitment

A convenience sample of caregivers was recruited from January 2022 to August 2022 via a variety of strategies including: (1) advertisements on websites, social media pages (e.g. Facebook, Twitter, Instagram), and/or newsletters of non-profit organisations for people with CKD and/or caregivers across the UK (e.g. Kidney Care UK, National Kidney Federation, the Global Kidney Foundation); (2) advertisements on a study Facebook and Twitter page; (3) an article published in the Spring 2022 issue of the free magazine produced by Kidney Care UK (Kidney Matters); and (4) a member of the research team speaking in an episode of a podcast (Diary of a Kidney Warrior). Paid Facebook advertisements were used for approximately four weeks (June—July 2022). Examples of study advertisements can be found in Additional file 2.

### Procedure

#### Informed consent

Potential participants who clicked the link to the survey were provided with study information and contact information for the research team. Informed consent was obtained electronically via Qualtrics before potential participants were able to proceed to the survey.

#### Survey

Potential participants providing informed consent were presented with brief screening questions to confirm eligibility (Additional file 3). Individuals were only able to complete the survey if they answered ‘yes’ to all screening questions.

The survey comprised four sections: (1) caregiver sociodemographic characteristics (16 items); (2) care recipient sociodemographic characteristics (12 items); (3) self-help intervention preferences (16 items with 27 sub-items); and (4) caregiver mental health (21 items). See Additional file 3 for full details of survey items.

The survey was comprised of 18 pages, in addition to three pages with (1) survey information and informed consent, (2) brief screening questions, and (3) end of survey thank-you message. The end of survey message informed participants they could contact the research team to receive a summary of the study results. On average, the number of items per page was 5 (range: 1 – 21). Items were presented in a fixed order, and a progress bar was displayed. Some adaptive questioning was used and is described below. All survey questions were voluntary. Participants were able to change/review answers using a back button. Survey functionality was tested by the research team. Within Qualtrics, a setting was activated to prevent multiple submissions from the same IP address, and cookies were used to allow participants to return to the survey if not completed in one sitting. Incomplete surveys (i.e. not submitted) were stored for one week before automatic deletion. IP addresses were not stored to ensure participant anonymity.

##### CBT self-help intervention preferences

Participants were presented with a text-based description of CBT self-help interventions prior to responding to questions regarding their intervention preferences. CBT self-help interventions were described to participants as a programme to support psychological wellbeing that could help caregivers cope with common emotional problems. Wider characteristics of CBT self-help interventions such as content, length, format, support options, were then described (see Additional file 3 for full description).

Adaptive questioning was used in two places within this section of the survey. Participants who indicated they were extremely unlikely to use a CBT self-help intervention if they experienced mental health difficulties were not shown any further survey items about intervention preferences. Additionally, participants who indicated they would not want to receive support from a trained professional were not shown survey items related to intervention support preferences.

##### Caregiver mental health

The 21-item Depression, Anxiety, and Stress Scale (DASS-21) [42] was used to measure depression, anxiety, and stress among caregivers. The DASS-21 is composed of three subscales to measure depression, anxiety, and stress with good internal consistency (Cronbach’s alpha above 0.7) [42].

### Data analysis

Descriptive statistics (means with standard deviations, proportions, and/or frequencies) were used to summarize caregiver and care recipient characteristics, preferences towards CBT self-help interventions, and their mental health. UK education levels were converted to International Standard Classification of Education (ISCED) levels [43]. For each subscale of the DASS-21, data was imputed for participants missing data for one item by replacing the item’s missing value with the mean of the values reported for all other items within the subscale [44]. Participants missing data for more than one item within a subscale were excluded from analysis of the relevant subscale. Quantitative analyses were conducted in RStudio Version 3.6.2 (RStudio, Boston, MA), with data from free-text survey items analysed by grouping similar comments into categories.

### Patient and public involvement

Patient and public involvement is reported following the Guidance for Reporting Involvement of Patients and the Public 2 – short form (Additional file 4) [45]. To ensure materials were easy to understand and acceptable to potential participants, public contributors, caregivers of people with CKD (*n* = 2), provided written feedback via email on (1) recruitment materials; (2) the participant information sheet; and (3) the survey. Feedback provided by public contributors resulted in concrete changes to study materials including: (1) removal of researcher photo from recruitment materials and information sheet; (2) modification of images used in recruitment materials to be more representative of the diversity of individuals who may care for someone with CKD; (3) reduced length of the information sheet by removing repetitive information; and (4) changes to survey questions and instructions to improve clarity.

## Results

### Recruitment

Ninety-two organisations were contacted regarding dissemination of survey recruitment materials to their networks with n ≈ 26 sharing the survey advertisement via Facebook (n ≈ 17), Twitter (n ≈ 12), the organisation’s website (n ≈ 7), newsletters (n ≈ 16), online forum (*n* = 1), and magazine (*n* = 1). Numbers are estimates as organisations may have shared survey advertisements without our knowledge and advertisements were not always posted on publicly available pages/newsletters. Posts regarding the survey from the study’s Facebook and Twitter pages were shared/re-tweeted approximately 5 and 13 times, respectively. Paid Facebook advertisements generated 1468 clicks. During the period when Facebook advertisements were active, 14 individuals submitted a survey, with 9 meeting inclusion criteria. However, we cannot know with certainty these participants were recruited via Facebook advertisements as it is possible they came across survey advertisements posted elsewhere.

### Participant characteristics

**Fig. 1:**
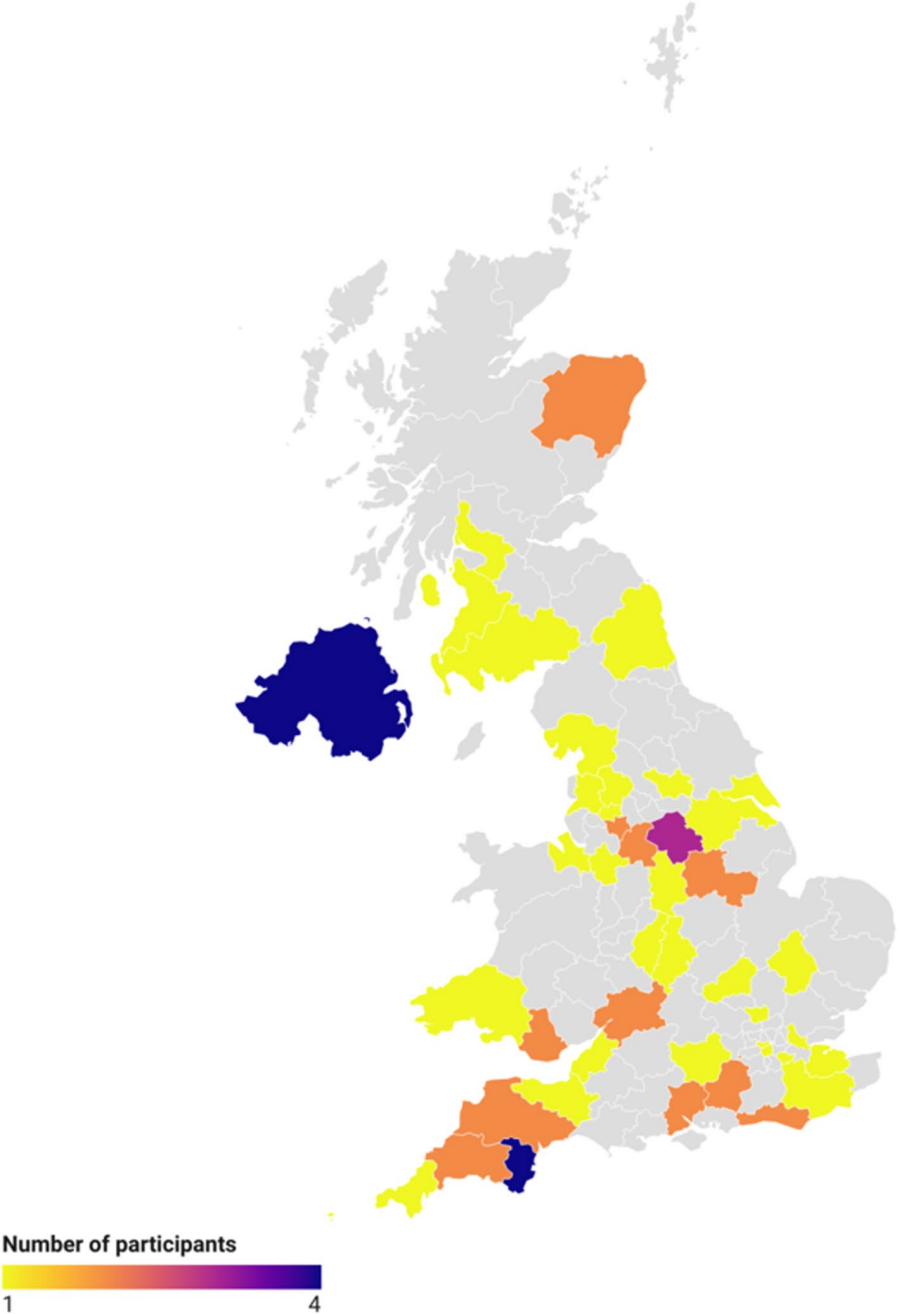
Map of participants’ geographic location in the UK (*n* = 63). Image created using Datawrapper [46]

A total of 65 participants completed the survey, with the majority being female (85%) with a mean age of 56, who were either retired (45%) or employed full-time (35%), living in England (85%) with a white ethnic background (97%; Table 1 and Fig. 1).

**Table 1:** Caregiver characteristics (*n* = 65)

Characteristic	Number	Percentage (%)
Age in years, mean (SD) [range]	56 (13) [28–82]	NA
Gender		
Female	55	85
Male	10	15
Country		
England	51	78
Northern Ireland	5	8
Scotland	5	8
Wales	4	6
Ethnic group		
White	63	97
Asian/Asian British	1	2
Mixed/multiple ethnic background	1	2
Black/African/Caribbean/Black British	0	0
Other	0	0
Prefer not to say	0	0
Relationship status		
Married/in a relationship	57	89
Divorced/separated	4	6
Single, never married	1	2
Widowed	1	2
Prefer not to say	1	2
Missing	1	2
Employment status		
Retired	29	45
Full-time	23	35
Part-time	10	15
Not employed	2	3
On sick leave	1	2
Highest level of education		
ISCED 6 or higher	30	46
ISCED 5	9	14
ISCED 3	19	29
No qualifications	1	2
Other	5	8
Prefer not to say	1	2
How well they are currently coping		
Very well	8	12
Well	25	38
Neither well nor not well	22	34
Not well	10	15
Not very well	0	0
DASS-21		
Depression, mean (SD)^a^	11 (10)	NA
Anxiety, mean (SD)^a^	7 (6)	NA
Stress, mean (SD)	14 (8)	NA

*Abbreviations*: *DASS-21* Depression, Anxiety and Stress Scale – 21 items, *ISCED* International Standard Classification of Education, *SD* Standard deviation

^a^Data was only available for 64 participants

### Caregiving situation

Information about the care recipient and caregiving situation are presented in Table 2. The majority of participants had been providing informal care to a male (77%) spouse or partner (74%) with a mean age of 53, for an average of 8 years. Participants were often the only person providing informal care to the care recipient, providing support with an average of 8 informal care activities (SD = 4). Few participants had assistance from formal (i.e. paid) in-home caregivers or received Carer’s Allowance. Additionally, some participants were providing informal care to someone else in addition to the person with CKD.

**Table 2:** Care recipient characteristics and description of the caregiving situation (*n* = 65)

Characteristic	Number	Percentage (%)
Care recipient		
Age in years, mean (SD) [range]^a^	53 (16) [18–77]	NA
Gender		
Female	15	23
Male	50	77
Approximate time since diagnosis in years, mean (SD) [range]^b^	14 (11) [1–42]	NA
Type of kidney condition(s)^c^		
Chronic kidney disease	64	98
Transplant	11	17
Missing	1	2
Type of treatment(s)^d^		
Kidney replacement therapy	45	69
Regular monitoring	42	65
Medication for their kidney condition	41	63
Medication for the disease causing the kidney condition	23	35
Cancer treatment	1	2
No treatment	1	2
Other	6	9.2
Terminally ill		
Yes	1	2
No	64	98
Number of other chronic conditions		
0 to 2	41	63
3 to 5	24	37
Receipt of informal care from anyone else		
Yes	9	14
No	55	85
Missing	1	2
Receipt of formal home care		
Yes	10	15
No	53	82
Missing	2	3
Caregiving role		
Time in caregiving role in years, mean (SD) [range]^a^	8 (8) [0.8 – 41]	NA
Caregiver’s relationship with the care recipient		
Spouse/partner	48	74
Parent	9	14
Child	4	6
Other family member	2	5
Friend	1	2
Other	1	2
Living with care recipient		
Yes	52	80
No^e^	13	20
Frequency of face-to-face contact with care recipient in a typical week		
Daily	55	85
A few days a week	7	11
Not seen every week	3	5
Informal care activities^d^		
Emotional support	54	83
Attending medical appointments with the care recipient	51	78
Running errands	48	74
Cleaning/gardening	44	68
Cooking	42	65
Managing symptoms	41	63
Driving	35	54
Communicating with medical care team	34	52
Organising medical care (e.g. making appointments)	27	42
Assist with home treatments	25	38
Managing finances	24	37
Assisting with medications	22	34
Getting around care recipient’s home	22	34
Bathing/showering	17	26
Wound care	16	25
Caring for access port or dialysis catheter	14	22
Getting dressed	11	17
Other	7	11
Receipt of Carer’s Allowance		
Yes	2	3
No	62	95
Missing	1	2
Presence of dependent children		
Yes	26	40
No	35	54
Missing	4	6
Provision of informal care to others		
Yes^f^	25	38
No	38	58
Missing	2	3

*Abbreviations*: *SD* Standard deviation

^a^Data available for 64 participants

^b^Data available for 63 participants

^c^Type of kidney condition was self-described by the caregiver. Care recipients with transplants were assumed to have chronic kidney disease as well, if not specified. Chronic kidney disease was further described by some participants with the following information: polycystic kidney disease (*n* = 9), IgA nephropathy (*n* = 7), Allports (*n* = 2), kidney cancer (*n* = 1), and various other conditions/causes (*n* = 14). Kidney failure and/or CKD stage 5 was mentioned by 25 participants

^d^Participants could select multiple responses

^e^Of 13 participants who did not live with the care recipient, 12 responded that they lived an average of 43 miles (SD = 85) away, spending an average of 1.1 h (SD = 1.8) travelling to the care recipient’s residence

^f^Those with other informal care responsibilities also cared for their parent(s) (*n* = 9), child(ren) (*n* = 7), grandchild(ren) (*n* = 2), other family member(s) (*n* = 9), friend (*n* = 1), neighbour (*n* = 1), and/or spouse/partner (*n* = 1)

### Caregiver mental health

Participants were experiencing mild depressive symptoms with a mean score of 11 (SD = 10) on the depression sub-scale of the DASS-21 (Table 1). Mean anxiety and stress scores were normal (Table 1). Classification of symptom severity showed that 58%, 38%, and 46% of participants were experiencing at least mild symptoms of depression, anxiety, and stress respectively (see Additional file 5 for categorical classification of DASS-21 scores). This included participants who reported severe or extremely severe symptoms of depression (16%), anxiety (9%), and stress (28%; Additional file 5).

### CBT self-help intervention preferences

Approximately half of the participants reported they were likely or extremely likely to use a CBT self-help intervention if experiencing mental health difficulties (Table 3). Four participants were extremely unlikely to use a CBT self-help intervention, and therefore not shown further questions related to intervention preferences. Of the sub-sample (*n* = 61) that proceeded to questions regarding intervention preferences, most had not used a self-help intervention before.

**Table 3:** Self-help intervention use and preferences (*n* = 61)

Characteristic	Number	Percentage (%)
Likelihood of using CBT self-help intervention (*n* = 65)		
Extremely likely	1	2
Likely	30	46
Neutral	15	23
Unlikely	15	23
Extremely unlikely	4	6
Previous use of a mental health self-help intervention		
Yes	18	30
No	41	67
Unsure	1	2
Prefer not to say	1	2
When information about the self-help intervention should be available		
At diagnosis	30	49
At start of a new treatment	14	23
During treatment	10	16
Other	6	10
Missing	1	2
Who should provide information about the intervention^a^		
Kidney patient or caregiver organisation	32	52
Doctor	30	49
Nurse	28	46
Peer	27	44
Support Group	23	38
Prefer to receive information via mail, email or paper information sheet	22	36
Psychologist/Counsellor	16	26
Social Worker	1	2
Other	1	2
Missing	1	2
When would the caregiver start using the intervention		
At diagnosis	26	43
At start of a new treatment	9	15
During treatment	19	31
Other	6	10
Missing	1	2
Who would the caregiver like to work on the intervention with		
A mixture of the options below	16	26
With the person I care for	14	23
Alone	13	21
Not sure	9	15
With other caregivers	8	13
Missing	1	2
How should content be made available		
All content available at all times	50	82
Time-released modules	10	16
Missing	1	2
How should content be presented^a^		
Video with experts	44	72
Text	31	51
Images	22	36
Video with animation	20	33
Audio	18	30
Video with actors	7	11
Missing	1	2
What device would the caregiver use to access the intervention^a^		
Computer	39	64
Smartphone	35	57
Tablet	27	44
Missing	1	2
Prefer intervention with support provided		
Yes	38	62
No	12	20
Unsure	11	18
Support preferences (*n* = 49)		
Where should intervention support be provided if it occurred in-person		
Own home	14	29
Community (e.g. local library, community centre)	10	20
GP’s practice	8	16
Psychological health service	6	12
Kidney/satellite unit	6	12
Hospital	2	4
Other	3	6
Who should provide support for the intervention		
Trained professional at a kidney patient or caregiver organisation	20	41
Trained professional at the kidney unit/hospital unit where the person you care for receives medical treatment	14	29
Peer	5	10
Psychologist/Counsellor	5	10
Nurse	2	4
Other	3	6
Social Worker	0	0

^a^Participants could select multiple responses

Participants preferred information about the intervention to be provided at diagnosis via a paper or electronic information sheet and/or from one of a variety of stakeholders (e.g. community organisations, healthcare professionals, peers), with one participant preferring information to come from the kidney unit (i.e. regardless of the specific professional background of the person providing information). One participant preferred social workers to provide intervention information. Participants preferred to use the intervention at diagnosis, highlighting that beginning a new treatment can be a challenging time to begin a new intervention. Comments from participants highlighted the importance of ensuring caregivers have access to information and can use the intervention at any time given caregivers may need support at different times. Caregivers preferred to work through the intervention with the care recipient (i.e. dyadic intervention) or to have a mixture of options regarding who they work through the intervention with (i.e. flexibility to work on intervention alone, with care recipient, and/or with peers). Further details regarding intervention preferences can be found in Table 3.

**Fig. 2:**
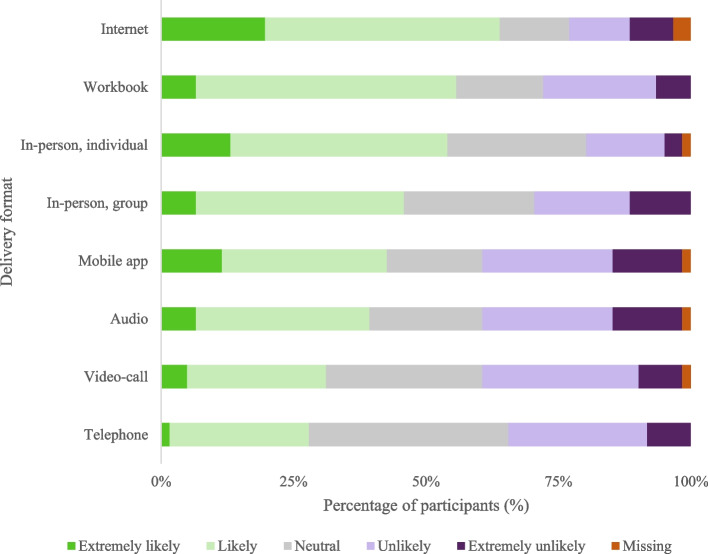
Intervention delivery format preferences (*n* = 61). Percentages represent how likely it was that participants would use an intervention with the indicated delivery format

Intervention delivery format preferences varied (Fig. 2, with numeric data in Additional file 6), with participants most likely to use an intervention that was internet-based, a workbook, or an individual in-person intervention. Participants were least likely to use an intervention delivered via telephone, or video-call.

#### Intervention content

**Fig. 3:**
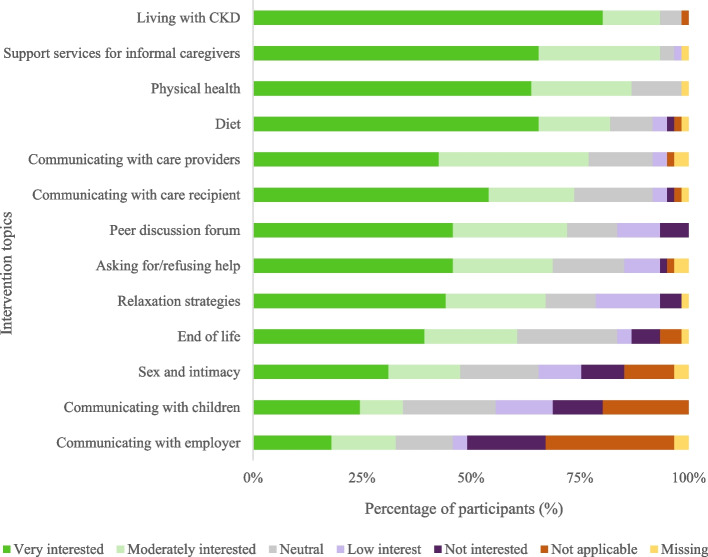
Caregiving related content preferences (*n* = 61). Percentages represent how interested participants were in each intervention topic

Participants preferred all intervention content to be available at once, rather than time-released (Table 3). Presentation of intervention content via videos with experts and text was preferred (Table 3). Participants were interested in all caregiving related topics suggested for inclusion to supplement CBT techniques and exercises (Fig. 3, with numeric data in Additional file 6), especially living with CKD, caregiver support services, caregiver’s physical health (e.g. sleep, exercise), and diet. Topics of least interest were communication with the caregiver’s employer, and communication with children, which were not applicable to all participants.

Participants described additional topics of interest including (1) finances and financial support; (2) how to prioritise themselves while caregiving (e.g. making time for themselves, how to not feel guilty for practicing self-care); (3) strategies to express feelings without negatively impacting the care recipient; (4) information about CKD (e.g. understanding CKD and treatments, planning for dialysis, life after transplant); (5) strategies to cope and live with challenges and changes due to CKD (e.g. unable to travel, changes to future life plans, feelings of fear, living with someone experiencing fatigue, coping with sudden changes, coping with being a potential kidney donor, strategies if transplant donor or recipient dies, or kidney is rejected); and (6) self-help resources for the care recipient and strategies for the caregiver to encourage the care recipient to access support. Information about support services for caregivers was a response option in the survey, however, participants provided further comments showing an interest in community and social support, information on where to seek help if experiencing difficulties with the medical care team, and information on where caregivers can access medical advice for the care recipient.

#### Intervention support

Over half of participants preferred a supported intervention (Table 3). Twelve participants did not want to receive support with a self-help intervention and were not asked further questions about intervention support preferences.

**Fig. 4:**
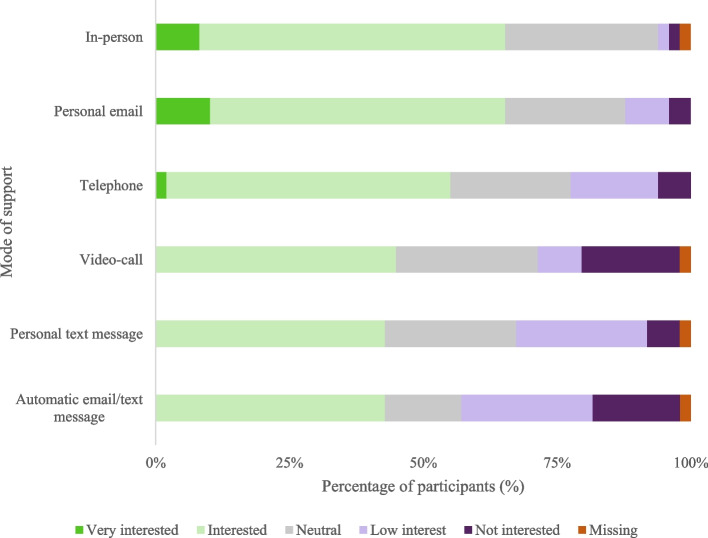
Intervention support mode preferences (*n* = 49). Percentages represent how interested participants were in receiving support provided via the indicated delivery mode

The majority of participants were most interested in support provided in-person or via a personal email (Fig. 4, with numeric data in Additional file 6). Support modes with the least interest were automatic emails/text messages, and personal text messages. Participants preferred to receive support from a trained professional at a community organization for kidney patients and/or caregivers, or a trained professional at the kidney unit (Table 3), with one participant commenting that a trained professional at the kidney unit would be preferable if support was received while at the kidney unit for the care recipient’s treatment (i.e. not involving an extra visit). If support was provided in-person, the caregiver’s home or a community-based setting were preferred locations (Table 3), with one comment stressing the importance of support being local and easy to get to. One participant commented receiving in-person support would be challenging due to difficulties/cost of finding someone who can take care of the care recipient to attend appointments, and lack of privacy.

#### General comments regarding CBT self-help interventions

Participants provided additional comments emphasising intervention delivery flexibility as essential given preferences can change over time as the caregiving situation changes. Digital intervention delivery modes (e.g. website, smartphone app, video-call) were viewed positively as strategies to improve access and overcome COVID-19 restrictions. Opportunities to receive support were important to facilitate discussion of intervention content, and for caregivers to receive reassurance and validation. Space to express negative emotions and receive acknowledgement without being directed to focus on the positive was valued. Finally, tailoring the intervention to caregivers of people with CKD, with support provided by someone knowledgeable about CKD, was viewed as essential to ensure the intervention would be impactful and relevant.

## Discussion

This study explored the preferences of caregivers of people with CKD regarding self-help interventions that support caregivers’ mental health in order to inform the future development of an intervention tailored towards their needs and preferences. A sample of sixty-five caregivers indicated a preference for an internet, workbook or in-person self-help intervention, containing information relevant for caregivers (e.g. living with CKD, support services), with intervention support provided in-person or via email by a trained professional at a community organisation. Key characteristics of this sample and identified self-help intervention preferences are discussed further below.

### Sample characteristics & representativeness

Participants were primarily women with a mean age of 56, and white ethnic background. Population-level data on caregivers of adults with CKD are not available, however, participant characteristics can be compared with caregivers in the UK more generally. Based on a sample of caregivers identified in the UK Census, just over half were women [47], and the largest proportion were aged 45 to 59 [48]. From the same Census, the majority of caregivers had a white ethnic background, and 10% had other ethnic backgrounds, with the majority having an Indian, Pakistani or Black Caribbean ethnic background [49]. Taken together, this suggests women caregivers with a white ethnic background may have been over-represented in this study.

A sub-group of participants cared for other family members (e.g. parent, child), and/or were working (full- or part-time) in addition to providing informal care to someone with CKD. These responsibilities can add additional stress and time-constraints [50], making flexible and accessible interventions essential. Future studies should explore whether there are additional delivery and content preferences to consider to ensure this subgroup of caregivers could engage in a CBT self-help intervention.

### Caregiver mental health

Prevalence of symptoms of depression, anxiety, and stress among caregivers of people with CKD was 58%, 38%, and 46% respectively, supporting the need to provide mental health support. Few other studies have examined the prevalence of mental health problems of caregivers of adults with CKD in the UK, however, studies from other countries have found similar findings, with prevalence of depressive symptoms between 30–60% [15, 17]. Prevalence of depressive symptoms in this sample was slightly higher compared to other caregiver populations, with meta-analyses showing depressive symptom prevalence rates of approximately 42%, 31%, and 40% among caregivers of people with cancer, dementia and stroke, respectively [51–53]. This is of particular interest given the greater focus on mental health interventions for cancer and dementia caregiver populations [20, 23, 54, 55], despite similar, if not higher, levels of mental health problems among caregivers of people with CKD. Findings further suggest the prevalence of depressive symptoms among caregivers is higher than in the general adult population, where prevalence of depressive symptoms is 17% in the UK [56].

### CBT self-help intervention preferences

CBT self-help interventions were an acceptable approach to supporting caregivers’ mental health, with a preference for an internet, workbook or individual in-person intervention with support provided in-person or via email by a trained professional at a community organisation. Interventions for caregivers of people with CKD described in research literature are primarily in-person, group-based programmes [20, 22]. Only 46% of participants indicated an interest in group-based interventions, and only 13% indicated an interest in working through the intervention exclusively with other caregivers. Additionally, interventions described in the literature are typically delivered by researchers, psychiatric nurses, or other healthcare professionals with experience in kidney care [20, 22]. Although caregivers expressed the importance of professionals providing support being knowledgeable about CKD, delivery of interventions by a nurse, psychologist or a trained professional at the kidney unit was preferred by only 4%, 10%, and 29% of participants, respectively. Our findings suggest existing interventions may not align with caregiver preferences.

There was strong interest for interventions to be delivered by trained professionals at community organisations. Community organisations in the UK (e.g. Kidney Care UK, National Kidney Federation, Popham Kidney Support) currently provide support to people living with CKD and their informal caregivers, such as information about CKD (e.g. information booklets, telephone helpline), and individual and group-based support delivered by psychologists and/or peers in different formats (e.g. telephone, in-person) [57–60]. However, to our knowledge CBT self-help interventions for caregivers are not available via community organisations. Provision of mental health support by community organisations may reduce stigma and could improve access to mental health support among individuals who would not seek care within a mental health service, an effect observed with community-based mental health outreach for older adults [61]. Additionally, kidney patient community organisations have a high level of expertise regarding CKD, which would allow them to provide support sensitive to the unique challenges caregivers experience. Future research may want to explore the capacity and resource/training needs within community organisations to implement and deliver CBT self-help interventions for caregivers.

Caregivers wanted information about the intervention to be available at all stages of the caregiving trajectory given caregivers may experience different support needs at different times. Caregiver support provision could be facilitated through regular monitoring of caregiver wellbeing given support needs can change according to the illness trajectory of the care recipient [62, 63], however this requires further research among caregivers of people with CKD. Information about the intervention should be available from a variety of stakeholders including health and social care professionals (e.g. community organization, doctor, nurse), peers, and support groups. Engagement of stakeholders early in intervention development is essential to ensure stakeholders are willing to have a role in implementing a CBT self-help intervention for caregivers. This aligns with the new MRC framework which places stakeholder engagement as a core component which should present during all phases of intervention development and evaluation [37]. Additionally, stakeholder engagement and buy-in is one of many factors that can facilitate intervention implementation [64, 65].

Caregivers indicated interest in most suggested intervention topics, including living with CKD, support services for caregivers, physical health, and diet. A review of information needs among caregivers of people with end-stage kidney disease [66] found that caregivers had a number of unmet information needs and generally did not receive enough information about practical aspects of caring for someone with CKD or understanding the condition. Available information was considered generic and not tailored to the needs of caregivers of people with CKD [66]. Tailoring interventions, in terms of content and delivery format, could be a way to meet caregiver’s varied needs, and appears important to enhance acceptability [67–69]. Internet-based interventions provide a way to tailor both intervention content and how content is delivered (e.g. audio, video, text) [70]. Flexibility in intervention content delivery has been reported as desirable during intervention development for different caregiving populations [71, 72]. As intervention development continues, methods to tailor and personalize the intervention should be explored as a strategy to enhance intervention acceptability.

### Recruitment

Despite use of varied recruitment approaches, survey participation was low. Caregiver recruitment is challenging, with data from a recent systematic review showing recruitment to randomized controlled trials of interventions for caregivers of people with CKD ranged from 38 to 105 participants [20]. In addition to common reasons for non-participation in research (e.g. lack of interest), caregivers report additional barriers such as (1) not identifying as a caregiver; (2) lack of time; and (3) caregiving responsibilities [73–75]. Although the sample size in this study was small, other caregiver surveys utilizing similar recruitment strategies, but including caregivers of adults with any health condition recruited 226 to 229 participants [76, 77]. Considering this study focused on a specific group of caregivers, the sample size may have been expected without employing recruitment approaches involving direct caregiver contact. Studies directly contacting caregivers via the research team and/or healthcare professionals have found this approach more effective compared to social media recruitment, possibly given the ability to form a relationship between potential participants and recruitment staff [73, 78].

### Patient and public involvement

Involvement of two public contributors resulted in a number of changes to study materials that improved recruitment materials, and clarified information presented within the survey. However, public involvement within this study was limited to a single feedback opportunity, and involvement was at the consultation level, with many decisions regarding study design already made by the research team. Collaborative involvement throughout the study design phase, may have resulted in further changes to study materials which could have impacted recruitment and study outcomes. Additionally, advertisement of the public involvement opportunity was limited, and may have benefitted from wider circulation to increase awareness of the involvement opportunity.

### Limitations

This study has several limitations. First, results may not be generalisable for three reasons (1) recruitment relied on convenience sampling, and based on sample characteristics (discussed above) our sample is unlikely to be representative of caregivers of adults with CKD throughout the UK; (2) the online format of the survey means we likely recruited caregivers with at least some level of digital literacy, which may have impacted results, particularly regarding intervention delivery format preference; and (3) the sample size is fairly small. Second, caregivers were asked about hypothetical intervention preferences based on a general description of CBT self-help interventions. It is possible that if participants were presented with a concrete intervention to relate their feedback to, preferences could change. Third, a deeper understanding of intervention preferences and the reasons for those preferences may have been facilitated through a mixed-methods approach.

Despite these limitations, to our knowledge this is the first study to explore CBT self-help intervention preferences of caregivers of people with CKD. Results provide initial insights into intervention preferences which can facilitate further intervention development. Future intervention development work will focus on developing a programme theory [39], selecting an appropriate framework to guide further intervention development [39], and will ideally engage caregivers and other stakeholders in a co-design process [36, 79, 80] to further inform content, format, and delivery methods.

## Conclusions

CBT-based self-help interventions appear to be an acceptable approach to providing psychological support to caregivers of people with CKD. Results indicated an internet, workbook, or individual in-person intervention supplemented with in-person or email support from a trained professional at a community organisation knowledgeable about CKD would meet the preferences of many caregivers. Although the intended application of the findings is to inform the development of a CBT self-help intervention, identified preferences could be applied to any intervention being developed for caregivers of people with CKD. Intervention tailoring could be used to better meet caregivers’ diverse preferences regarding intervention delivery format and content. Future work should seek to engage with caregivers and other stakeholders (e.g. healthcare professionals, staff from community organisations) to further inform the development process, and implementation frameworks (e.g. the Consolidated Framework for Implementation Research [81]) should be used to explore factors that may influence implementation of the CBT self-help intervention into practice.

## Supplementary Information

**Additional file 1. **Checklist for Reporting Results of Internet E-Surveys (CHERRIES).

**Additional file 2. **Examples of recruitment materials.

**Additional file 3. **Survey.

**Additional file 4. **Guidance for Reporting Involvement of Patients and the Public 2 – short form (GRIPP2-SF).

**Additional file 5. **DASS-21 categories.

**Additional file 6. **Data corresponding to Figure 2-4.

## Data Availability

The datasets used and/or analyzed during the current study are available in the Zenodo repository [82].

## Abbreviations

CBT: Cognitive Behavioural Therapy
CKD: Chronic Kidney Disease
DASS-21: Depression, Anxiety, and Stress Scale – 21 items
IP address: Internet Protocol address
MRC: Medical Research Council
UK: United Kingdom
