# Supplementary material for: Cognitive behavioural therapy self-help intervention preferences among informal caregivers of adults with chronic kidney disease: an online cross-sectional survey
Source: BMC Nephrol. 2023 Jan 4;24:4. doi: 10.1186/s12882-022-03052-7 (PMC9812545; doi:10.1186/s12882-022-03052-7)
Supplement: Supplementary file 2 — Additional file 2. Examples of recruitment materials. [file 12882_2022_3052_MOESM2_ESM.pdf]

Additional file 2 – Examples of recruitment materials

Are you a family member, partner, or friend of someone living with a kidney condition?

We are designing a support programme for family, partners, and friends who help someone with a kidney condition. What would you want in a programme like this? Tell us in our online survey!

What do we mean by "helping someone with a kidney condition"? It could mean something like...

Helping with their medical care or talking with their care team

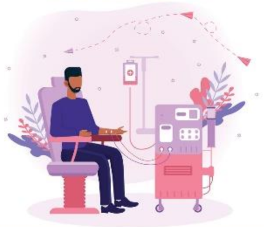

Helping around the house like cooking or cleaning

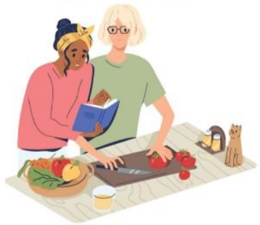

Listening to them if they are worried or stressed

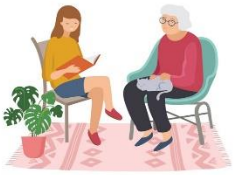

If you are interested in participating please use the QR code or this link to go to our survey!
https://exetercles.eu.qualtrics.com/jfe/form/SV_725d9rMSoarmOr4

Need more information? Contact Chelsea at:
c.coumoundouros@exeter.ac.uk

ENTWINE
INFORMAL CARE

Who can participate? Anyone who is...

- At least 18 years old
- Living in the UK
- A family member, partner, or friend who is helping someone with a kidney condition who is also at least 18 years old

UNIVERSITY OF EXETER

The survey will take around 15-20 minutes to complete.

If you are someone who is living with a kidney condition, please pass this information on to any family member, partner, or friend who provides you with help and support.

Thank you!

Are you a family member, partner, or friend of someone living with a kidney condition?

We are designing a support programme for family, partners, and friends who help someone with a kidney condition. What would you want in a programme like this? Tell us in our online survey!

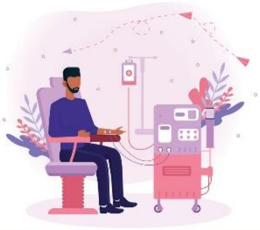

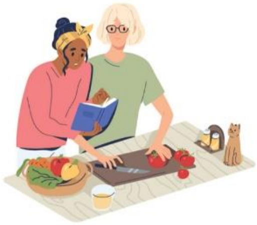

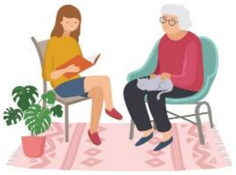

If you are interested in participating please use the QR code or this link to go to our survey!
https://exetercles.eu.qualtrics.com/jfe/form/SV_725d9rMSoarmOr4

Need more information? Contact Chelsea at: c.coumoundouros@exeter.ac.uk

ENTWINE
INFORMAL CARE

Who can participate? Anyone who is...

- At least 18 years old
- Living in the UK
- A family member, partner, or friend who is helping someone with a kidney condition who is also at least 18 years old

UNIVERSITY OF EXETER

The survey will take around 15-20 minutes to complete.

If you are someone who is living with a kidney condition, please pass this information on to any family member, partner, or friend who provides you with help and support.

Thank you!

Are you a family member, partner, or friend of someone living with a kidney condition?

We are designing a support programme for family, partners, and friends who help someone with a kidney condition. What would you want in a programme like this? Tell us in our online survey!

What do we mean by “helping someone with a kidney condition”? It could mean something like…

Helping with their medical care or talking with their care team

Helping around the house like cooking or cleaning

Listening to them if they are worried or stressed

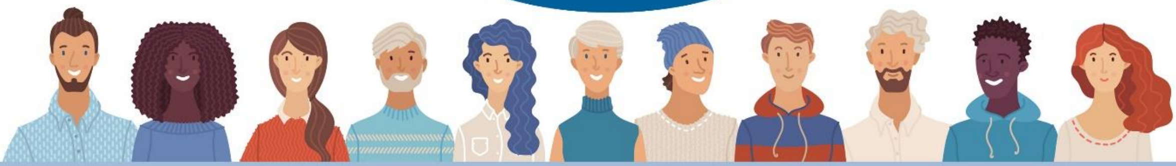

If you are interested in participating please use the QR code or this link to go to our survey!
https://exetercles.eu.qualtrics.com/jfe/form/SV_725d9rMSoarmOr4

Need more information? Contact Chelsea at:
c.coumoundouros@exeter.ac.uk

ENTWINE INFORMAL CARE

UNIVERSITY OF EXETER

Who can participate? Anyone who is…

- At least 18 years old
- Living in the UK
- A family member, partner, or friend who is helping someone with a kidney condition who is also at least 18 years old

The survey will take around 15-20 minutes to complete.

If you are someone who is living with a kidney condition, please pass this information on to any family member, partner, or friend who provides you with help and support.

Thank you!

Are you a family member, partner, or friend of someone living with a kidney condition?

We are designing a support programme for family, partners, and friends who help someone with a kidney condition. What would you want in a programme like this? Tell us in our online survey!

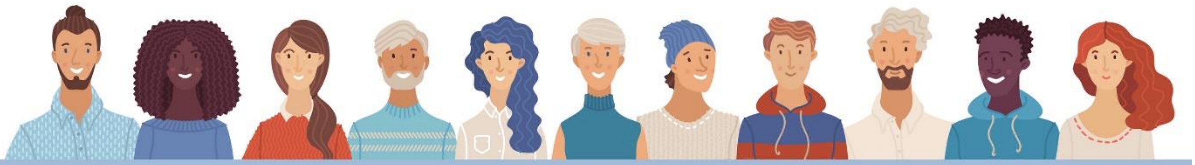

If you are interested in participating please use the QR code or this link to go to our survey!
https://exetercles.eu.qualtrics.com/jfe/form/SV_725d9rMSoarmOr4

Need more information? Contact Chelsea at:
c.coumoundouros@exeter.ac.uk

ENTWINE
INFORMAL CARE

Who can participate? Anyone who is...

- At least 18 years old
- Living in the UK
- A family member, partner, or friend who is helping someone with a kidney condition who is also at least 18 years old

UNIVERSITY OF
EXETER

The survey will take around 15-20 minutes to complete.

If you are someone who is living with a kidney condition, please pass this information on to any family member, partner, or friend who provides you with help and support.

Thank you!

Example of paid Facebook adverts

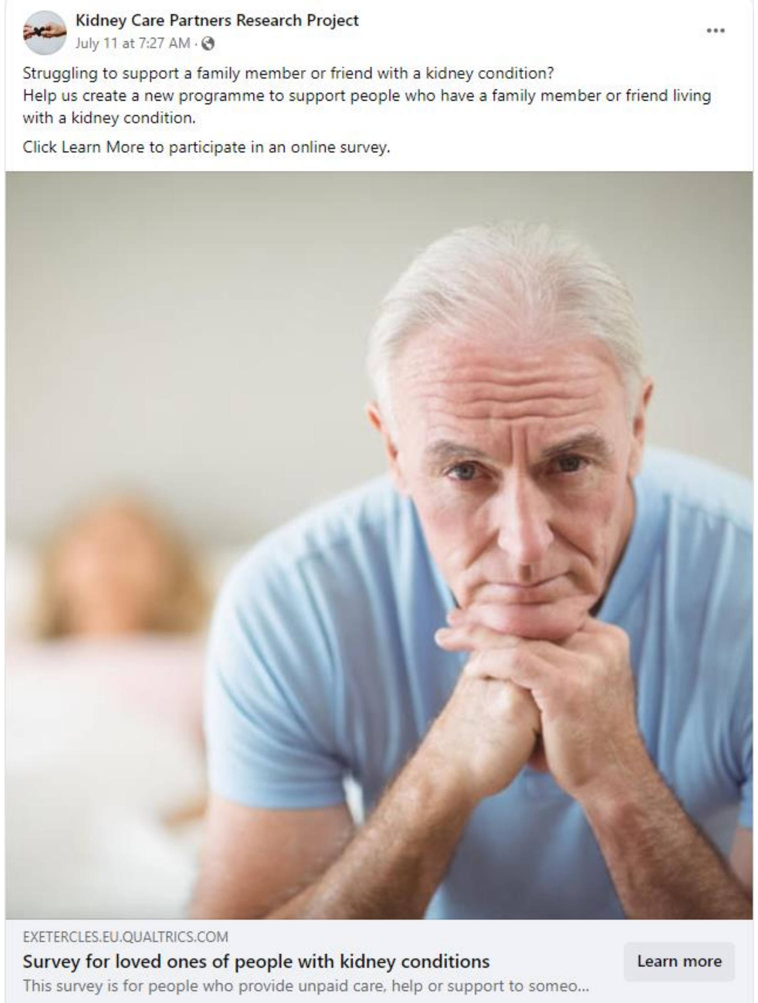

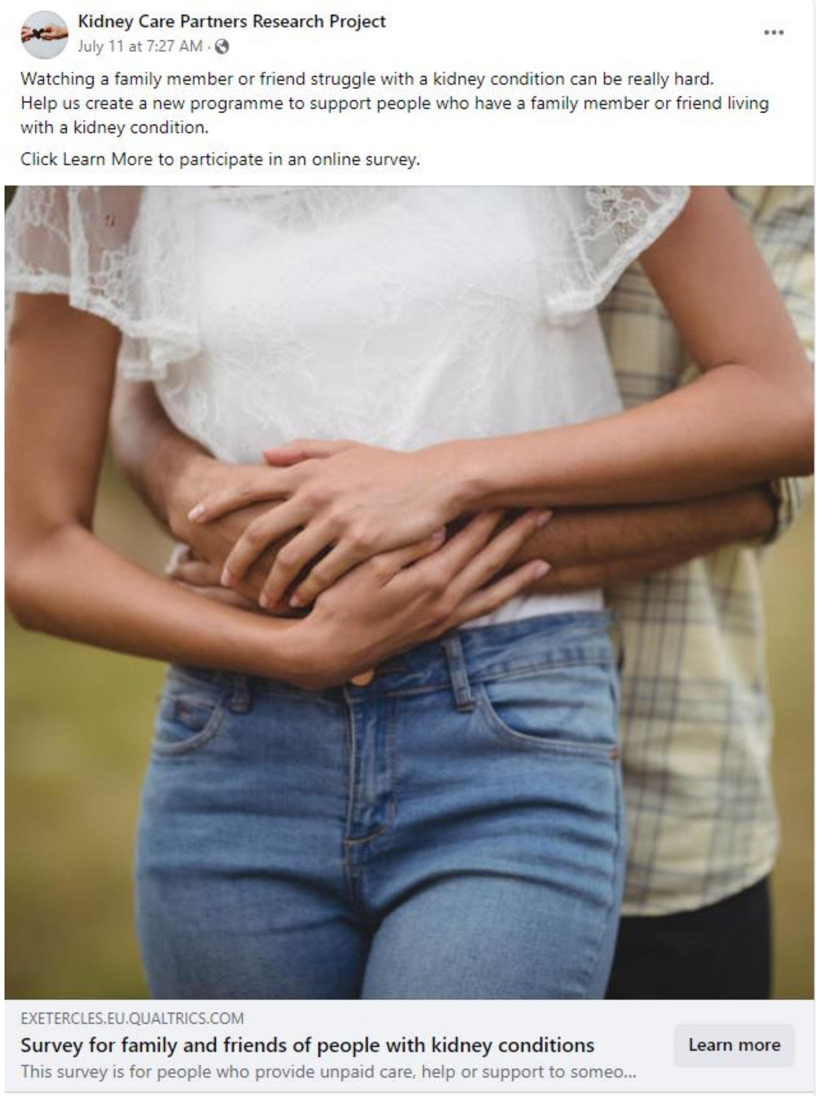

Kidney Care Partners Research Project
July 11 ·

Want to help create a support programme for loved ones of someone living with a kidney condition?

Click Learn More to participate in an online survey.

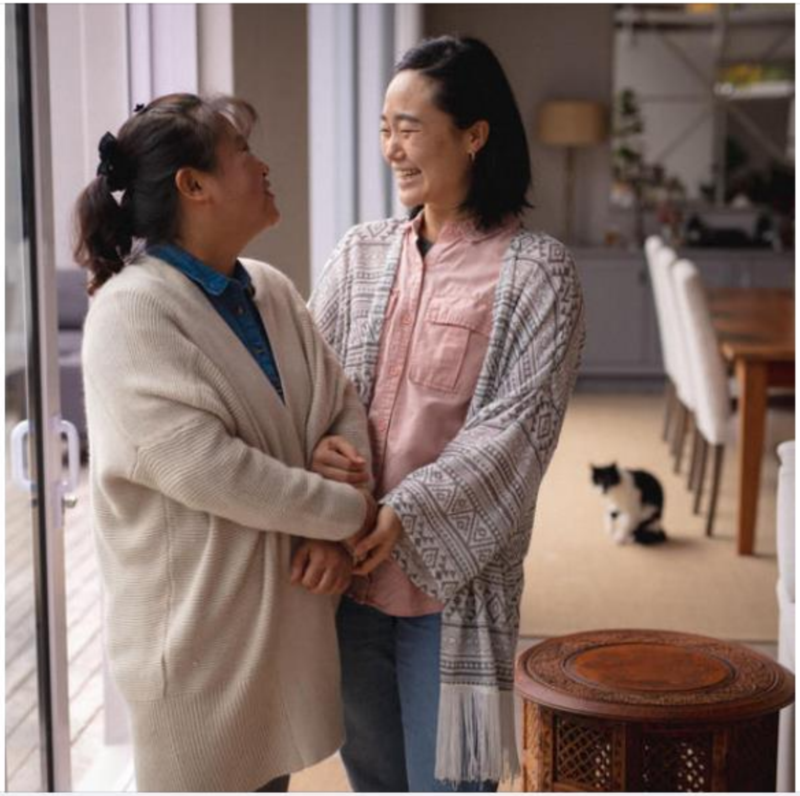

EXETERCLES.EU.QUALTRICS.COM

Survey for family and friends of people with kidney conditions

This survey is for people who provide unpaid care, help or support to someo...

Learn more

Kidney Care Partners Research Project
July 2 at 8:29 PM ·

Want to help create a support programme for loved ones of someone living with a kidney condition?
Do you have a loved one who is living with a kidney condition?

Click Learn More to participate in an online survey.

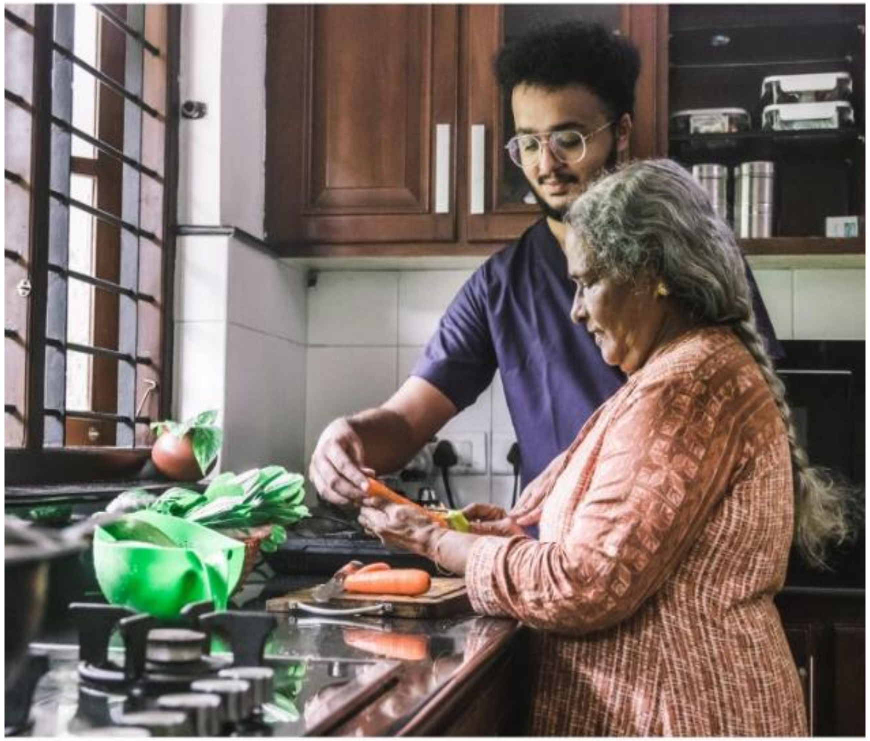

EXETERCLES.EU.QUALTRICS.COM

Survey for family and friends of people with kidney conditions

Learn more
