# Supplementary material for: Cognitive behavioural therapy self-help intervention preferences among informal caregivers of adults with chronic kidney disease: an online cross-sectional survey
Source: BMC Nephrol. 2023 Jan 4;24:4. doi: 10.1186/s12882-022-03052-7 (PMC9812545; doi:10.1186/s12882-022-03052-7)
Supplement: Supplementary file 3 — Additional file 3. Survey. [file 12882_2022_3052_MOESM3_ESM.pdf]

Additional file 3: Survey

Part A: Is this study for you?

First, we would like to make sure this survey is for you.

1. **Are you aged 18 years or older?** *Please tick one option.*
   - ☐ Yes
   - ☐ No

2. **Do you live in the United Kingdom?** *Please tick one option.*
   - ☐ Yes
   - ☐ No

3. **Are you currently providing any kind of unpaid care, help, and support to someone with a kidney condition?** *See the box below for some examples of unpaid care activities. Please tick one option.*
   - ☐ Yes
   - ☐ No

Unpaid care, help and support can include things like:

- **Household activities** (e.g. cleaning, cooking, shopping) you would not have done if the person you care for was in good health
- Activities related to your friend/family member's **medical care** (e.g. accompanying them to medical appointments, getting prescriptions for them or helping them with their medical care, communicating with their care team)
- **Emotional/psychological support** (e.g. helping them with feelings of worry, stress, or pain)

4. **Is the person with a kidney condition you care for aged 18 years or older?** *Please tick one option.*
   - ☐ Yes
   - ☐ No

[If no is selected for any of these questions, participant is not eligible for study and survey skips to end]

Part B: About You

Now we would like to know about you.

1. **How old are you?** ____________________ years

2. **What is your gender?** *Please tick one option.*
   - ☐ Female
   - ☐ Male
   - ☐ Other
   - ☐ Prefer not to say

3. **Where are you located?** *Please tick one option.*
   - ☐ England
   - ☐ Wales
   - ☐ Scotland
   - ☐ Northern Ireland
   - ☐ Prefer not to say

4. **What is the first half (3 to 4 characters) of your postcode?** (e.g. if your postcode is EX4 4QJ, you would write EX4 in the space provided) ________________________________

5. **What is your ethnic group?** *Please tick one option.*

   White

   - ☐ English/Welsh/Scottish/Northern Irish/British
   - ☐ Irish
   - ☐ Irish traveler or Romany gypsy
   - ☐ Any other white background

   Mixed/multiple ethnic background

   - ☐ White and Black Caribbean
   - ☐ White and Black African
   - ☐ White and Asian
   - ☐ Any other mixed/multiple ethnic background

   Asian/Asian British

   - ☐ Indian
   - ☐ Pakistani
   - ☐ Bangladeshi
   - ☐ Chinese
   - ☐ Any other Asian background

   Black/African/Caribbean/Black British

   - ☐ African
   - ☐ Caribbean
   - ☐ Any other Black/African/Caribbean background

   Other ethnic group

   - ☐ Arab
   - ☐ Any other ethnic group

   - ☐ Prefer not to say

UNIVERSITY OF EXETER

ENTWINE INFORMAL CARE

6. **What is your relationship status?** *Please tick one option.*
   - ☐ Married/In a relationship
   - ☐ Single, never married
   - ☐ Divorced/Separated
   - ☐ Widowed
   - ☐ Prefer not to say

7. **How many children do you have that you support in any way?** *For example, children you prepare meals for, financially support etc.* ______________________children

8. **What is your current employment status?** *Please tick one option.*
   - ☐ Full-time
   - ☐ Part-time
   - ☐ Homemaker
   - ☐ Retired
   - ☐ Student
   - ☐ On sick-leave
   - ☐ Not employed
   - ☐ Prefer not to say

9. **What is your highest level of qualification?** *Please tick one option.*
   - ☐ Apprenticeship (e.g., trade, advanced, foundation, modern, Level 4-7)
   - ☐ GCSEs or equivalent
   - ☐ AS, A level, or equivalent
   - ☐ NVQ Level 1, 2, or 3, BTEC National or General, City and Guilds Craft or Advanced Craft
   - ☐ Foundation degree, HND, HNC, NVQ Level 4 and above
   - ☐ Bachelor's degree or higher
   - ☐ I have other qualifications, but I do not know what they are equivalent to
   - ☐ I do not have any qualifications
   - ☐ Prefer not to say

10. **How long have you been providing care to someone with a kidney condition?**

_____________________ years _____________________ months

11. Which of the activities listed below do you help the person you care for with? *These activities may be things you only occasionally help with or you may help with this all the time. Please tick ALL that apply.*

- ☐ Bathing/showering
- ☐ Caring for their access port or dialysis catheter
- ☐ Cleaning or gardening where they live
- ☐ Communicating with their medical care team
- ☐ Cooking for them
- ☐ Driving them where they need to go
- ☐ Getting dressed
- ☐ Going to medical appointments with them
- ☐ Getting around where they live (e.g. helping them with stairs or walking to a different room)
- ☐ Listening and comforting them when they feel worried or stressed
- ☐ Managing their finances (e.g. helping them pay bills)
- ☐ Managing any symptoms or side-effects they are experiencing
- ☐ Organising their medical care (e.g. making appointments with their care team, arranging homecare assistance)
- ☐ Treatments they receive at home (e.g. at-home dialysis)
- ☐ Taking medications (e.g. organising what medication they should take, giving them an injection)
- ☐ Running errands for them (e.g. shopping for groceries, clothes)
- ☐ Wound care
- ☐ Other(s), please specify:

12. How are you currently coping with providing this care? *Please tick one option.*

Not very well	Not well	Neither well nor not well	Well	Very well
☐	☐	☐	☐	☐

13. Does the person you care for receive unpaid care from anyone else (e.g. unpaid care from another family member or friend)? *Please tick one option.*

- ☐ Yes
- ☐ No
- ☐ Not sure

UNIVERSITY OF EXETER

ENTWINE INFORMAL CARE

14. **Does the person you care for receive any formal care in their home (e.g. homecare assistance via the council, a homecare agency, renal home therapy team, district nurse or you employ your own carer)?** *Please tick one option.*
    - ☐ Yes
    - ☐ No
    - ☐ Not sure

15. **Do you receive the Carer's Allowance?** *Please tick one option.*
    - ☐ Yes
    - ☐ No
    - ☐ Not sure

16. **Who else do you provide unpaid care to other than the person with a kidney condition?** *Please tick ALL that apply.*
    - ☐ There is no one else I provide unpaid care to
    - ☐ Spouse/partner
    - ☐ Child(ren)
    - ☐ Parent(s)
    - ☐ Grandchild(ren)
    - ☐ Other, please specify:
    - ☐ Other family member(s)
    - ☐ Friend
    - ☐ Colleague
    - ☐ Neighbour

Part C: About the Person You Care For

*We would like to know more about the **person with a kidney condition** that you provide unpaid care, help and support to. You may be providing care to more than one person, please answer the question based on the **person with a kidney condition** you care for the most.*

2. **How old is the person you care for?** ______________________years

3. **What is the gender of the person you care for?** *Please tick one option.*
    - ☐ Female
    - ☐ Male
    - ☐ Other
    - ☐ Prefer not to say

4. **What is your relationship with the person you care for?** *Please tick one option.*
   ***You** are their…*
    - ☐ Spouse/Partner
    - ☐ Child
    - ☐ Parent
    - ☐ Other family member
    - ☐ Other, please specify:____
    - ☐ Friend
    - ☐ Neighbour
    - ☐ Colleague

UNIVERSITY OF EXETER

ENTWINE INFORMAL CARE

5. **Do you live in the same house/apartment as the person you care for?** *Please tick <u>one</u> option.*
   - ☐ Yes *[if yes is selected, survey skips to question 8]*
   - ☐ No

6. **Approximately how far away do you live from the person you care for?**

   ____________________________miles

7. **Approximately how long does it typically take you to arrive at their place of residence?**

   ________________________________minutes

8. **How often do you see the person you care for face-to-face in a typical week?** *Please tick <u>one</u> option.*
   - ☐ Daily
   - ☐ A few days a week
   - ☐ Once a week
   - ☐ I do not see them every week

The next few questions are about the health and well-being of the person you care for.

9. **Approximately when was the person you care for first diagnosed with a kidney condition?**

   _______________________(MM-YYYY)

10. **What type of kidney condition(s) do they have?** *Please list all kidney conditions the person you care for has.*

11. **What type of treatment(s) are they currently receiving?** *Please tick <u>ALL</u> that apply.*
    - ☐ No treatment
    - ☐ Receiving regular monitoring and follow-up with a healthcare provider (GP, nephrologist etc.)
    - ☐ Medications for their kidney condition (e.g. iron infusions, EPO injections, tablets for bones)
    - ☐ Medications for the disease causing kidney problems (e.g. insulin for diabetes, immunosuppressants for vasculitis)
    - ☐ Kidney replacement therapy (e.g. haemodialysis, peritoneal dialysis, kidney transplant)
    - ☐ Cancer treatment
    - ☐ Other, please specify:

12. **Is the person you care for terminally ill?** *Someone who is terminally ill has an illness that cannot be cured and is expected to live for 6 months or less. Please tick one option.*
    - ☐ Yes
    - ☐ No

13. **How many other chronic conditions, not including their kidney condition, does the person you care for have that are expected to last or have already lasted 6 months or more?** *Some examples of chronic conditions are diabetes, heart conditions, high blood pressure, asthma, arthritis. Please tick one option.*
    - ☐ 0 to 2 conditions
    - ☐ 3 to 5 conditions
    - ☐ 6 to 9 conditions
    - ☐ 10 or more conditions

Part D: Self-Help Programme Preferences

The following questions relate to your preferences about self-help programmes to help improve psychological well-being. You may not have used a self-help programme before, so try to imagine what you would prefer if you were to use one.

What are self-help programmes for psychological well-being?

Self-help programmes for psychological well-being are used to help people cope with difficulties that can be commonly experienced by people who provide care, help, and support to people living with a kidney condition (e.g. low mood, feeling down, sad, anxious, worried, or stressed).

Self-help programmes include specific evidence-based psychological techniques, for example based on cognitive behavioural therapy, and activities (e.g. thinking about what is important to you, planning tasks, setting goals for yourself) that are designed to be completed over several weeks.

Self-help programmes can be delivered in different formats like printed workbooks, websites, smartphone apps, over the phone, video call, or in person. Self-help programmes can be used on your own, however, a trained professional can be available to offer support and guidance to use the materials.

1. **If you were to experience difficulties with your psychological well-being** (e.g. low mood, feeling down, sad, anxious, worried, or stressed)**, how likely is it that you would use a self-help programme?** *Please tick one option.*
    - ☐ Extremely unlikely *[if extremely unlikely is selected, survey skips to next section (DASS-21)]*
    - ☐ Unlikely
    - ☐ Neutral
    - ☐ Likely
    - ☐ Extremely likely

2. **Have you ever sought help from a self-help programme for your psychological well-being in the past?** *Please tick one option.*
   - ☐ Yes
   - ☐ No
   - ☐ Not sure
   - ☐ Prefer not to say

3. **Self-help programmes can be delivered in different formats. How likely would you be to use each of the different self-help programme formats listed below?** *Tick one option for each format presented.*

	Extremely unlikely	Unlikely	Neutral	Likely	Extremely likely
**Audio-based** programme (e.g. audiobook with recorded information and exercises you listen and follow along to)	☐	☐	☐	☐	☐
**Internet-based** programme (e.g. information and exercises delivered via a website)	☐	☐	☐	☐	☐
**In-person programme** and **meet one-on-one** with a trained professional to work through the self-help programme	☐	☐	☐	☐	☐
**In-person programme** and **meet as a group** with a trained professional and others who care and support someone with a kidney condition to work through the self-help programme	☐	☐	☐	☐	☐
**Smartphone app** (e.g. information and exercises delivered via a smartphone app)	☐	☐	☐	☐	☐
**Telephone-based** programme (e.g. phone call with a trained professional) to work through the self-help programme	☐	☐	☐	☐	☐

**Video-call** (e.g Skype, Zoom or Teams meeting with a trained professional) to work through the self-help programme	☐	☐	☐	☐	☐
**Workbook** (e.g. a printed booklet with information and exercises)	☐	☐	☐	☐	☐

4. **When would you most like to receive information about available self-help programmes?** *Please tick one option.*
   - ☐ When the person I care for was first diagnosed
   - ☐ At the start of a new treatment
   - ☐ During treatment
   - ☐ Other, please specify: ____

5. **Who would you most like to provide you with information about available self-help programmes?** *Please tick ALL option.*
   - ☐ Doctor
   - ☐ Kidney patient or caregiver organisation
   - ☐ Nurse
   - ☐ Peer (someone who has cared for a family member or friend with a kidney condition)
   - ☐ Prefer to have information via mail, email or paper information sheet
   - ☐ Psychologist/Counsellor
   - ☐ Social worker
   - ☐ Support group
   - ☐ Other, please specify: ____

6. **When would you most want to start using a self-help programme?** *Please tick one option.*
   - ☐ When the person I care for was first diagnosed
   - ☐ At the start of a new treatment
   - ☐ During treatment
   - ☐ Other, please specify: ____

7. **When using a self-help programme, who would you most like to work on the programme with?** *Please tick one option.*
   - ☐ On my own
   - ☐ With the person I care for (e.g. you go through the information and do activities together)
   - ☐ With other people who care for someone with a kidney condition
   - ☐ A mixture of the above options
   - ☐ Not sure

8. **When using a self-help programme, how would you most like content to be made available to you?** *Please tick <u>one</u> option.*
    - ☐ All content of the programme to be available at all times
    - ☐ Programme content to be released over time in sections (e.g. one section released each week)

9. **How would you like content to be presented?** *Please tick <u>ALL</u> that apply.*
    - ☐ Audio
    - ☐ Images
    - ☐ Text
    - ☐ Video with animation
    - ☐ Video with actors
    - ☐ Video with experts (e.g. psychologist, nurse, social worker)

10. **If the self-help programme was online, what device(s) would you use to access it?** *Please tick <u>ALL</u> that apply*
    - ☐ Computer (desktop or laptop)
    - ☐ Tablet
    - ☐ Smartphone

11. **Self-help programmes include information and specific evidence-based techniques to help people overcome difficulties with their psychological well-being. Self-help programmes can also include additional information that people might find helpful when providing care to those living with a kidney condition. What additional information you would like to be included in a self-help programme?** *Tick <u>one</u> choice that best reflects your level of interest for each topic.*

	Not Interested	Little interest	Neutral	Moderately interested	Interested	Not applicable
**Relaxation strategies**	☐	☐	☐	☐	☐	☐
**How to ask for help or refuse help from others** (e.g. family, friends, health and social care)	☐	☐	☐	☐	☐	☐
**Communication tips:** talking to the <u>person you care for</u>	☐	☐	☐	☐	☐	☐
**Communication tips:** talking to <u>children and young people</u> about kidney diseases	☐	☐	☐	☐	☐	☐
**Communication tips:** talking to <u>care providers</u>	☐	☐	☐	☐	☐	☐

**Communication tips:** talking to your boss	☐	☐	☐	☐	☐	☐
**Discussion forum** to share experiences with other people who care for someone with a kidney condition	☐	☐	☐	☐	☐	☐
**Information about support services** for people who provide unpaid care for someone with a kidney condition	☐	☐	☐	☐	☐	☐
**Information about physical health** issues you may have (e.g. sleep, exercise)	☐	☐	☐	☐	☐	☐
**Information about diet** (e.g. nutrition, recipes, foods to avoid)	☐	☐	☐	☐	☐	☐
**Information about sex and intimacy**	☐	☐	☐	☐	☐	☐
**Information about living with kidney conditions**	☐	☐	☐	☐	☐	☐
**Information about end of life and grief**	☐	☐	☐	☐	☐	☐

Are there any other topics you would like a self-help programme to cover?

You can use self-help programmes on your own. However, a trained professional can be available to offer support and guidance to use the self-help programme. The next few questions are about the type of support and guidance you would want if you were using a self-help programme.

12. **If using a self-help programme, would you like to receive support and guidance from a trained professional?** *Please tick one option.*
    - ☐ Yes, I would like to receive support and guidance from a trained professional
    - ☐ No, I would prefer to use a self-help programme on my own *[if this response is selected, survey skips to open text box for other suggestions at end of this section]*
    - ☐ Not sure

13. **If using a self-help programme, what type of support would you be interested in?** *Support refers to feedback and guidance you would receive whilst using the programme. Tick one option for each type of support presented.*

	Not interested	Little interest	Neutral	Interested	Very interested
**Automatic email or SMS** messages with reminders and encouragement	☐	☐	☐	☐	☐
**Personal email:** you would be contacted by email regularly by a trained professional	☐	☐	☐	☐	☐
**In-person:** you would regularly meet a trained professional face-to-face	☐	☐	☐	☐	☐
**SMS:** you would be contacted by text message regularly by a trained professional	☐	☐	☐	☐	☐
**Telephone:** you would be contacted regularly by a trained professional via telephone	☐	☐	☐	☐	☐
**Video-call:** you would be contacted regularly by a trained professional via video-call (such as Skype, Zoom or Teams)	☐	☐	☐	☐	☐

14. If support was provided in-person, where would you most like that support to be provided? *Please tick one option.*

- ☐ At a psychological health service
- ☐ At the hospital
- ☐ At your GP's practice
- ☐ At your own home
- ☐ At your renal/satellite unit
- ☐ Somewhere in your community (e.g. local library, community centre)
- ☐ Other, please specify: ____________________

15. Who would you most like to provide this support? *Please tick one option.*

- ☐ Nurse
- ☐ Peer (someone who has cared for a family member or friend with a kidney condition)
- ☐ Psychologist/Counsellor
- ☐ Social worker
- ☐ Trained professional at a kidney patient or caregiver organisation
- ☐ Trained professional at the renal unit/hospital unit where the person you care for receives medical treatment
- ☐ Other, please specify: ____________________

Is there anything else you would like us to know about your preferences for self-help programmes?

Part E: How you are feeling

Please read each statement and tick that option that indicates how much the statement applied to you over the past week. There are no right or wrong answers. Do not spend too much time on any statement.

	Did not apply to me at all	Applied to me to some degree, or some of the time	Applied to me a considerable degree, or a good part of the time	Applied to me very much, or most of the time
1) I found it hard to wind down	☐	☐	☐	☐
2) I was aware of dryness of my mouth	☐	☐	☐	☐
3) I couldn’t seem to experience any positive feelings at all	☐	☐	☐	☐
4) I experienced breathing difficulty (e.g. excessively rapid breathing, breathlessness in the absence of physical exertion)	☐	☐	☐	☐
5) I found it difficult to work up the initiative to do things	☐	☐	☐	☐
6) I tended to over-react to situations	☐	☐	☐	☐
7) I experienced trembling (e.g. in the hands)	☐	☐	☐	☐
8) I felt that I was using a lot of nervous energy	☐	☐	☐	☐
9) I was worried about situations in which I might panic and make a fool of myself	☐	☐	☐	☐
10) I felt that I had nothing to look forward to	☐	☐	☐	☐
11) I found myself getting agitated	☐	☐	☐	☐
12) I found it difficult to relax	☐	☐	☐	☐
13) I felt down-hearted and blue	☐	☐	☐	☐
14) I was intolerant of anything that kept me from getting on with what I was doing	☐	☐	☐	☐
15) I felt I was close to panic	☐	☐	☐	☐
16) I was unable to become enthusiastic about anything	☐	☐	☐	☐
17) I felt I wasn't worth much as a person	☐	☐	☐	☐
18) I felt that I was rather touchy	☐	☐	☐	☐
19) I was aware of the action of my heart in the absence of physical exertion (e.g. sense of heart rate increase, heart missing a beat)	☐	☐	☐	☐
20) I felt scared without any good reason	☐	☐	☐	☐
21) I felt that life was meaningless	☐	☐	☐	☐
