# Supplementary material for: Cognitive behavioural therapy self-help intervention preferences among informal caregivers of adults with chronic kidney disease: an online cross-sectional survey
Source: BMC Nephrol. 2023 Jan 4;24:4. doi: 10.1186/s12882-022-03052-7 (PMC9812545; doi:10.1186/s12882-022-03052-7)
Supplement: Supplementary file 4 — Additional file 4. Guidance for Reporting Involvement of Patients and the Public 2 – short form (GRIPP2-SF). [file 12882_2022_3052_MOESM4_ESM.pdf]

Additional file 4: Guidance for Reporting Involvement of Patients and the Public 2 – short form (GRIPP2-SF)

Section and topic	Item	Location in text
1: Aim	Report the aim of PPI in the study	Pg 8
2: Methods	Provide a clear description of the methods used for PPI in the study	Pg 8
3: Study results	Outcomes—Report the results of PPI in the study, including both positive and negative outcomes	Pg 8
4: Discussion and conclusions	Outcomes—Comment on the extent to which PPI influenced the study overall. Describe positive and negative effects	Pg 8 & 20
5: Reflections/critical perspective	Comment critically on the study, reflecting on the things that went well and those that did not, so others can learn from this experience	Pg 20

Abbreviation: PPI: patient and public involvement

From Staniszewska, S., Brett, J., Simera, I., Seers, K., Mockford, C., Goodlad, S., Altman, D. G., Moher, D., Barber, R., Denegri, S., Entwistle, A., Littlejohns, P., Morris, C., Suleman, R., Thomas, V., & Tysall, C. (2017). GRIPP2 reporting checklists: tools to improve reporting of patient and public involvement in research. *BMJ, 358*, j3453. https://doi.org/10.1136/bmj.j3453
