# Supplementary material for: Cognitive behavioural therapy self-help intervention preferences among informal caregivers of adults with chronic kidney disease: an online cross-sectional survey
Source: BMC Nephrol. 2023 Jan 4;24:4. doi: 10.1186/s12882-022-03052-7 (PMC9812545; doi:10.1186/s12882-022-03052-7)
Supplement: Supplementary file 5 — Additional file 5. DASS-21 categories. [file 12882_2022_3052_MOESM5_ESM.pdf]

Additional file 5: DASS-21 categories

Table S1: Classification of severity of depressive symptoms from the DASS-21 (n = 64)

	Number	Percentage (%)
Normal (≤ 9)	27	42
Mild (10-13)	11	17
Moderate (14-20)	16	25
Severe (21-27)	4	6
Extremely severe (≥ 28)	6	9

Table S2: Classification of severity of anxiety symptoms from the DASS-21 (n = 64)

	Number	Percentage (%)
Normal (≤ 7)	40	63
Mild (8-9)	6	9
Moderate (10-14)	12	19
Severe (15-19)	3	5
Extremely severe (≥ 20)	3	5

Table S3: Classification of severity of stress symptoms from the DASS-21 (n = 65)

	Number	Percentage (%)
Normal (≤ 14)	35	54
Mild (15-18)	13	20
Moderate (19-25)	9	14
Severe (26-33)	8	12
Extremely severe (≥ 34)	0	0
