# Supplementary material for: Cognitive behavioural therapy self-help intervention preferences among informal caregivers of adults with chronic kidney disease: an online cross-sectional survey
Source: BMC Nephrol. 2023 Jan 4;24:4. doi: 10.1186/s12882-022-03052-7 (PMC9812545; doi:10.1186/s12882-022-03052-7)
Supplement: Supplementary file 6 — Additional file 6. Data corresponding to Figure 2-4. [file 12882_2022_3052_MOESM6_ESM.pdf]

Additional file 6: Data corresponding to Figure 2-4.

Table S1: Intervention delivery format preferences (n = 61). Data corresponding to Figure 2.

	Extremely likely n (%)	Likely n (%)	Neutral n (%)	Unlikely n (%)	Extremely unlikely n (%)	Missing n (%)
Internet	12 (20)	27 (44)	8 (13)	7 (11)	5 (8)	2 (3)
Workbook	4 (7)	30 (49)	10 (16)	13 (21)	4 (7)	0
In-person, individual	8 (13)	25 (41)	16 (26)	9 (15)	2 (3)	1 (2)
In-person, group	4 (7)	24 (39)	15 (25)	11 (18)	7 (11)	0
Mobile app	7 (11)	19 (31)	11 (18)	15 (25)	8 (13)	1 (2)
Audio	4 (7)	20 (33)	13 (21)	15 (25)	8 (13)	1 (2)
Video-call	3 (5)	16 (26)	18 (30)	18 (30)	5 (8)	1 (2)
Telephone	1 (2)	16 (26)	23 (38)	16 (26)	5 (8)	0

Table S2: Caregiving related content preferences (n = 61). Data corresponding to Figure 3.

	Very interested n (%)	Moderately interested n (%)	Neutral n (%)	Low interest n (%)	Not interested n (%)	Not applicable n (%)	Missing n (%)
Living with CKD	49 (80)	8 (13)	3 (5)	0	0	1 (2)	0
Support services for caregivers	40 (66)	17 (28)	2 (3)	1 (2)	0	0	1 (2)
Physical health	39 (64)	14 (23)	7 (11)	0	0	0	1 (2)
Diet	40 (66)	10 (16)	6 (10)	2 (3)	1 (2)	1 (2)	1 (2)
Communicating with care providers	26 (43)	21 (34)	9 (15)	2 (3)	0	1 (2)	2 (3)
Communicating with care recipient	33 (54)	12 (20)	11 (18)	2 (3)	1 (2)	1 (2)	1 (2)
Peer discussion forum	28 (46)	16 (26)	7 (11)	6 (10)	4 (7)	0	0
Asking for/refusing help	28 (46)	14 (23)	10 (16)	5 (8)	1 (2)	1 (2)	2 (3)
Relaxation strategies	27 (44)	14 (23)	7 (11)	9 (15)	3 (5)	0	1 (2)
End of life	24 (39)	13 (21)	14 (23)	2 (3)	4 (7)	3 (4.9)	1 (2)
Sex and intimacy	19 (31)	10 (16)	11 (18)	6 (10)	6 (10)	7 (11)	2 (3)
Communicating with children	15 (25)	6 (10)	13 (21)	8 (13)	7 (11)	12 (20)	0
Communicating with employer	11 (18)	9 (15)	8 (13)	2 (3)	11 (18)	18 (30)	2 (3)

Table S3: Intervention support mode preferences (n = 49). Data corresponding to Figure 4.

	Very interested n (%)	Interested n (%)	Neutral n (%)	Low interest n (%)	Not interested n (%)	Missing n (%)
In-person	4 (8)	28 (57)	14 (29)	1 (2)	1 (2)	1 (2)
Personal email	5 (10)	27 (55)	11 (22)	4 (8)	2 (4)	0
Telephone	1 (2)	26 (53)	11 (22)	8 (16)	3 (6)	0
Video-call	0	22 (45)	13 (27)	4 (8)	9 (18)	1 (2)
Personal text message	0	21 (43)	12 (24)	12(24)	3 (6)	1 (2)
Automatic email/text message	0	21 (43)	7 (14)	12 (24)	8 (16)	1 (2)
